# ENPP2 alleviates hypoxia/reoxygenation injury and ferroptosis by regulating oxidative stress and mitochondrial function in human cardiac microvascular endothelial cells

**DOI:** 10.1007/s12192-023-01324-1

**Published:** 2023-04-13

**Authors:** Guanhua Fang, Yanming Shen, Dongshan Liao

**Affiliations:** 1 Department of Cardiovascular Surgery, Union Hospital, Fujian Medical University, Key Laboratory of Cardio-Thoracic Surgery (Fujian Medical University), Fujian Province University, Fuzhou, 350001 Fujian China; 2 Fujian Medical University, Fuzhou, 350001 Fujian China

**Keywords:** Hypoxia/reoxygenation injury, ENPP2, Oxidative stress, Ferroptosis, Mitochondrial function

## Abstract

This study aimed to elucidate the molecular mechanisms of hypoxia/reoxygenation (H/R) injury in human cardiac microvascular endothelial cells (HCMECs) by regulating ferroptosis. H/R model was established with HCMECs and before the reperfusion, ferroptosis inhibitor ferrostatin-1 or ferroptosis inducer erastin was all administered. Wound-healing assay was performed to detect the migration ability of cells in each group, and the angiogenesis ability was determined by tube formation assay. The level of reactive oxygen species (ROS) was detected by flow cytometry. Transmission electron microscopy (TEM) was used to observe the state of mitochondria. The expressions of related proteins in HCMECs were assessed by Western blot. From the results, H/R injury could inhibit the migration and angiogenesis, induce the ROS production, and cause the mitochondrial damage of HCMECs. Ferroptosis activator erastin could aggravate H/R injury in HCMECs, while the ferroptosis inhibitor ferrostatin-1 could reverse the effects of H/R on HCMECs. Western blot results showed that H/R or/and erastin treatment could significantly induce ACSL4, HGF, VEGF, p-ERK, and uPA protein expression and inhibit GPX4 expression. The addition of ferrostatin-1 resulted in the opposite trend of the proteins expression above to erastin treatment. What is more, overexpression of ENPP2 markedly suppressed the damaging effect of H/R on HCMECs and reversed the effects of H/R or erastin treatment on the expression of related proteins. These results demonstrated a great therapeutic efficacy of ENPP2 overexpression in preventing the development of H/R injury through inhibiting oxidative stress and ferroptosis.

## Introduction

Myocardial ischemia refers to the pathophysiological state of abnormal myocardial energy metabolism due to insufficient blood supply and oxygen supply, resulting in the failure of the heart to maintain its normal function (Ambrose 2006). The incidence rate of myocardial ischemia accounts for the highest proportion in many pathogenesis associated with heart failure, which poses a great health threat to the patients with cardiovascular disease (Pagliaro, Cannata, Stefanini, and Bolognese 2020). At present, coronary artery reperfusion therapy is mainly used in clinic to effectively reduce myocardial ischemia injury and prevent further tissue damage, but the reperfusion process will also bring damage that cannot be ignored (Binder et al. 2015; B. Shi, Ma, Zheng 2019, Pan, and Lin 2019). Myocardial ischemia/reperfusion (I/R) is a pathological process characterized by insufficient oxygen supply and subsequent blood flow recovery that occurs in many organs and diseases with high morbidity and mortality (Zhang et al. 2022). I/R injury may be caused by pathological processes and/or secondary to surgical processes, and the original I/R injury is more severe (B. Shi et al. 2019). Additionally, the mechanisms contributing to the pathogenesis of I/R is complex, multifactorial, and highly integrated (Lejay et al. 2016). Consequently, preventing hypoxia- reoxygenation (H/R) injury and resolving its molecular mechanism are vital for improving the healing rate of myocardial I/R injury.

Cardiac microvascular endothelial cells (CMECs) are, by necessity, the cells affected in the cardiac microcirculation by I/R injury (Cui et al. 2016). Compared to vascular endothelial cells, CMECs are more sensitive to ischemic injury and have attracted much attention in preventing myocardial I/R injury (Singhal, Symons, Boudina, Jaishy, and Shiu 2010). Previous studies have shown that diversified abnormal responses and detrimental changes of CMECs were initiated by I/R, including excessive inflammation, reactive oxygen species (ROS) production and apoptosis, contributing to the progression of cardiac dysfunction (Y. Liu et al. 2014; Zhu et al. 2018).

Recent studies have demonstrated that ferroptosis and oxidative stress ferroptosis are closely related to the process of I/R injury. Oxidative stress is usually caused by uncontrolled high levels of ROS, resulting in imbalance of oxidation and antioxidant systems and cell and tissue damage (García, Zazueta, and Aguilera-Aguirre 2017; González-Montero, Brito, Gajardo, and Rodrigo 2018; Kandula et al. 2016). Since ROS induces multiple types of cell death, including apoptosis, necrosis, and pyroptosis, it is considered to have a major role in I/R injury of many organs (Li et al. 2018). Ferroptosis is an iron-dependent regulatory of cell death (Dixon et al. 2012), which has been found to have a role in many diseases and has been proposed as a new therapeutic strategy for I/R injury (She, Lan, Tian, and Tang 2020). There is evidence that inhibiting ferroptosis had the ability of alleviating diabetes I/R injury, which might give a therapeutic direction for myocardial ischemic disease (W. Li et al. 2020). Importantly, it is currently believed that ferroptosis is mainly related to abnormal toxicity of iron, lipid peroxidation, and mitochondrial dysfunction (Conrad et al. 2018). Moreover, researchers found that selenium can reduce cerebral I/R injury through affecting oxidative stress, ferroptosis, and mitochondrial fusion in vivo and in vitro (Y. Shi et al. 2022). However, whether myocardial I/R regulates human cardiac microvascular endothelial cell (HCMEC) injury through ferroptosis and the specific regulatory mechanism are still unclear.

Here, to address these unanswered questions, we constructed hypoxia/reoxygenation (H/R) cell model in vitro with HCMECs, combined with ferroptosis inducers or inhibitors to evaluate the H/R injury and ferroptosis in cell function, oxidative stress, and mitochondrial morphology, as well as further explored the molecular mechanism of H/R injury affecting HCMECs through ferroptosis.

## Methods

### Cell culture, model induction, and cell treatment

Human cardiac microvascular endothelial cells (HCMECs) were obtained from ScienCell Research Laboratories, Inc. and cultured in endothelial cell medium with 5 mmol/L glucose and 10% FBS (Gibco; Thermo Fisher Scientific, Inc.) at 37 °C with 5% CO2.

### Induction of H/R model

HCMECs were first cultured in glucose-free and serum-free endothelial cell medium and exposed to hypoxia environment at 37 °C (94% N_2_, 5% CO_2_, and 1% O_2_) for 12 h to induce hypoxia. Subsequently, the culture medium was replaced with DMEM medium containing 4.5 g/L glucose and supplemented with 10% FBS (Invitrogen, USA), 100 U/mL penicillin, and 100 U/mL streptomycin (Solarbio Science & Technology Co., Ltd., China), and the cells were cultured under normoxic conditions (95% air and 5% CO_2_) for 24 h at 37 °C to induce reoxygenation.

To explore the effect of ferroptosis, HCMECs were treated with ferrostatin-1 (1 μM) or erastin (10 μM) for 8 h before the H/R model induction.

### Adenovirus-mediated gene transfer into HCMECs

The adenovirus vector with ENPP2 gene overexpression was constructed by the manufacturer (Gene Pharma, China). HCMECs were seeded into 24-well culture dishes, and when they reached 50% confluence, cells were transfected with the ENPP2 overexpression adenovirus (Ad. ENPP2) or negative control adenovirus (Ad. null) at a multiplicity of infection of 100.

### Tube formation assay

The wells of the 96-well plate were coated with 50 μL of Matrigel® matrix basement membrane (Corning® 354234) and incubated for 30 min, and then HCMECs were seeded on the gel with an average of 1 × 10^4^/well. After incubation for 12 h, the tube formation of HCMECs was observed by a microscope.

### Wound-healing assay

For the wound-healing assay, HCMECs of each group were cultured to 100% confluence, and then a scratch was made using a pipet tip (10 μL). After that, HCMECs were cultured with serum-free endothelial cell medium. The wounds were photographed at 0 and 24 h, and the migration distance of the HUVECs was measured.

### Western blot

HCMECs from each treatment group were first lysed with RIPA lysis buffer. Then, the total protein concentration was evaluated with the protein assay kit (Beyotime Biotech, China). Total protein concentration was probed with primary antibodies: HGF (1:3000, ab178395; Abcam), GPX4 (1:5000, ab125066; Abcam), VEGF (1:5000, ab32152; Abcam), ACSL4 (1:10,000, ab155282; Abcam), p-ERK (1:1000, ab201015; Abcam), uPA (1:1000, ab218106; Abcam), ENPP2 (1:500, ab77104; Abcam), and GAPDH (1:5000, ab9485; Abcam), followed by detection with an ECL kit (Thermo Fisher Scientific, USA). Finally, the protein was quantitatively analyzed using chemidoc imaging system (Tanon, China) and ImageJ analysis software.

### ROS detection

According to the manufacturer’s optimized instructions, the intracellular levels of ROS in HCMECs from different treatment groups were analyzed by the probe of DCFH-DA, a ROS detection kit (Beyotime, China). The cells were collected and suspended in the diluted DCFH-DA (10 μM) and incubated for 20 min in a 37-°C cell incubator. After fully contacting the probe and the cells by reverse mixing, the cells were washed with serum-free cell culture solution to fully remove DCFH-DA. After collecting the cells, the samples loaded with the probe were detected by flow cytometry.

### Transmission electron microscopy (TEM)

In order to observe the damage of mitochondrial morphology in HCMECs, we performed TEM observation on cells in each group. Firstly, HCMECs were prefixed with 2% glutaraldehyde and then fixed with 1% osmium tetroxide. Next, the samples were dehydrated in ethanol containing 3% uranyl acetate and embedded in propylene oxide and epoxy resin overnight. Subsequently, the samples were polymerized into slices (70-nm thick) and stained with lead citrate. Eventually, an H-7650 transmission electron microscope (Hitachi) was used for testing and observation.

### Statistical analysis

All the data were analyzed with Prism 8.0 (GraphPad, USA), presented as mean ± SEM. For difference comparisons between groups, unpaired Student’s *t* test or one-way analysis of variance (ANOVA) was employed. *p* < 0.05 was indicated statistically significant.

## Results

### H/R injury inhibited the migration and angiogenesis of HCMECs by exacerbating ferroptosis

**Fig. 1:**
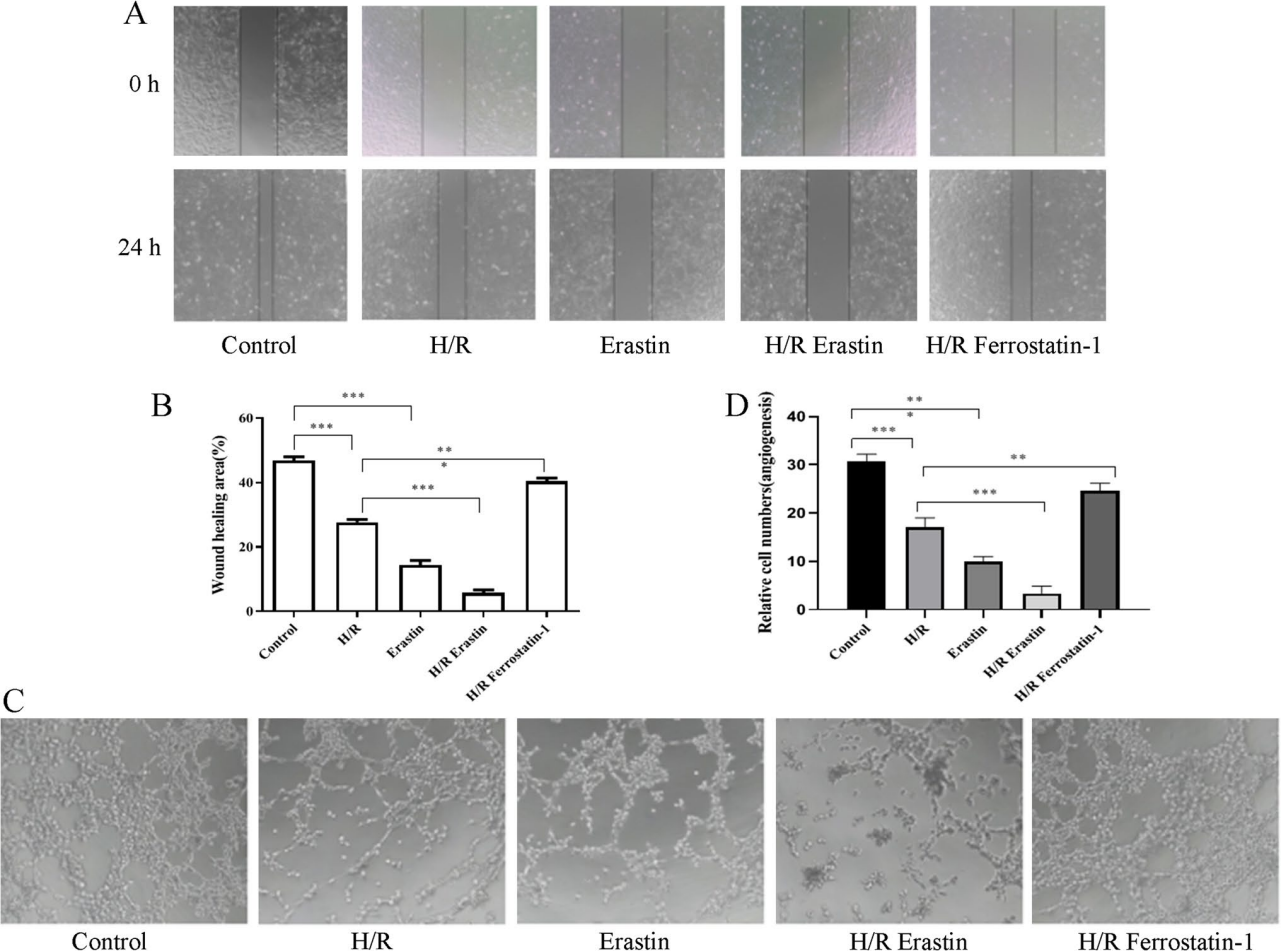
H/R injury inhibited the migration and angiogenesis of HCMECs by exacerbating ferroptosis**. **We first established H/R model with HCMECs, with treatment of ferroptosis activator, erastin, or ferroptosis inhibitor, ferrostatin-1, the group of which was named as: control, H/R, H/R erastin, and H/R ferrostatin-1. **A**, **B** The wound healing assay was used to detect the migration ability of HCMECs in each group. **C**, **D** The tube formation assay was used to detect the angiogenesis ability of HCMECs in each group. Data are presented as the mean ± SEM (*n* = 3). *, *p* < 0.05; **, *p* < 0.01; ***, *p* < 0.001

To explore the effect of H/R injury and ferroptosis on cellular function of HCMECs, HCMECs were exposed to H/R conditions along with erastin (ferroptosis activator) or ferrostatin-1 (ferroptosis inhibitor). Then, we detected the migration ability and angiogenesis ability of the cells in each group. The wound-healing assay revealed that compared with the control group, the migration ability was apparently restrained in H/R or erastin group, while it was reversed following ferrostatin-1 administration (Fig. 1A, B). As shown in Fig. 1C, tube formation ability of HCMECs was obviously elevated with H/R or/and erastin treatment, while the co-treatment of H/R and ferrostatin-1 induced the induction of angiogenesis (Fig. 1C, D). These results indicated that H/R might inhibit the migration and angiogenesis of HCMECs by regulating ferroptosis.

### H/R injury induced the oxidative stress and mitochondrial damage of HCMECs by exacerbating ferroptosis

**Fig. 2:**
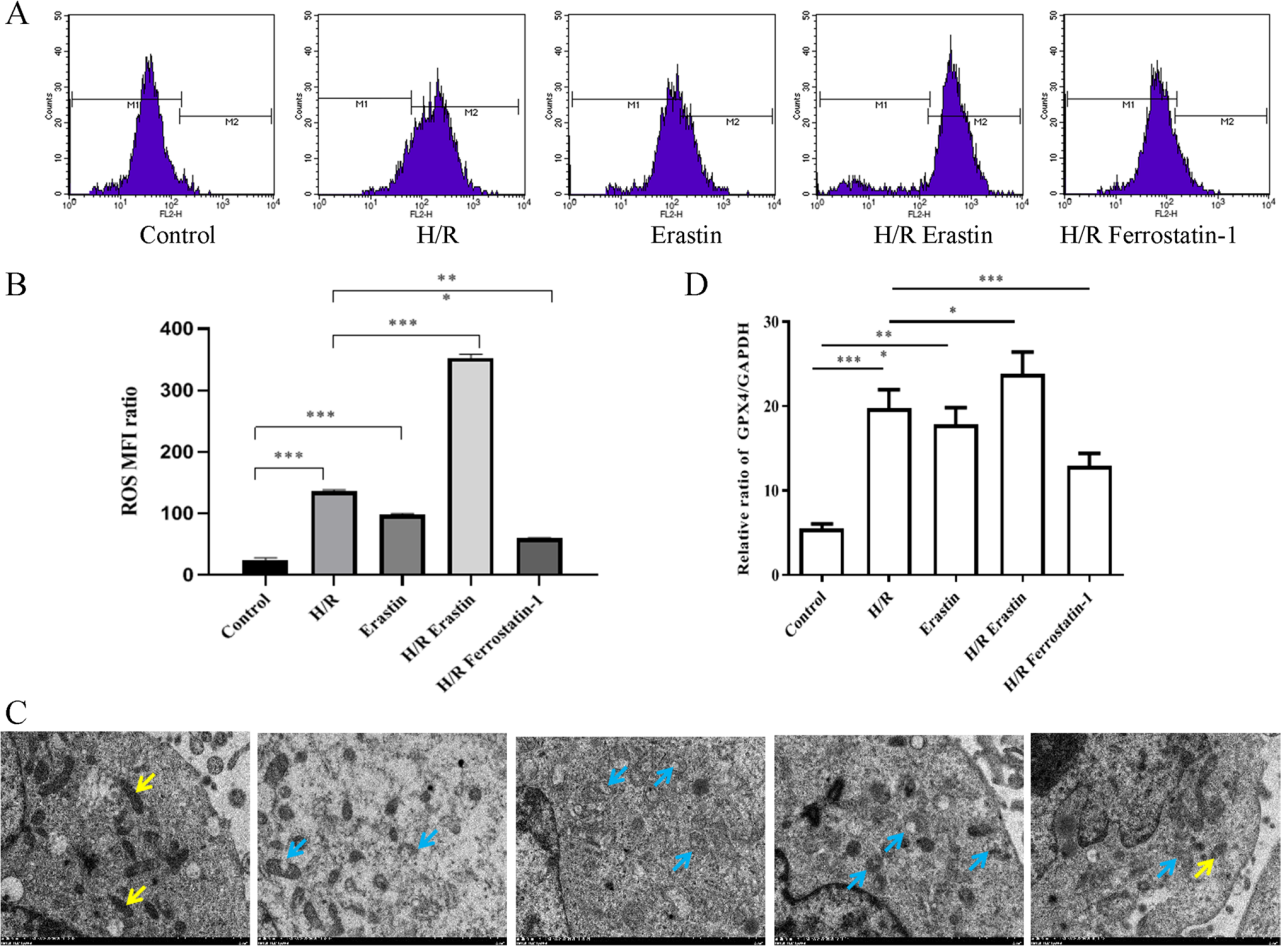
H/R injury and ferroptosis induced the oxidative stress and mitochondrial damage of HCMECs by exacerbating ferroptosis. **A**, **B **The production of cellular ROS was analyzed by flow cytometry.** C **TEM was used to observe the state of mitochondria in each group of cells; the yellow arrows indicate normal mitochondria and blue arrows indicate swollen cracked mitochondria.** D **Statistical analysis of swollen cracked mitochondria in each group. Data are presented as the mean ± SEM** (***n* **= **3). *, *p* **< **0.05; **, *p* **<** 0.01; ***, *p* **< **0.001

Studies have indicated that H/R injury was related to ferroptosis and mitochondrial dysfunction (Chen et al. 2022). Therefore, the next question to address was whether H/R stimulation could regulate oxidative stress and mitochondrial function by exacerbating ferroptosis in HCMECs. We first performed flow cytometry to detect the production of ROS in each group of HCMECs. The results revealed that in the presence of H/R condition or/and erastin treatment, the production of cellular ROS was apparently increased, while it was further decreased under H/R + ferrostatin-1 conditions compared with in H/R group (Fig. 2A, B). In addition, TEM was used to observe the state of mitochondria in each group of cells. There was an increase intracellular swelling, and breakage of mitochondria was observed in H/R condition or/and erastin treatment. Compared with this, there was a significant reduction of mitochondrial swelling and fragmentation in H/R + ferrostatin-1 group (Fig. 2C, D). These observations verified that H/R injury increased ROS production and accelerated mitochondrial damage by exacerbating ferroptosis.

### Mechanisms of H/R injury induced ferroptosis affecting the function of HCMECs

**Fig. 3:**
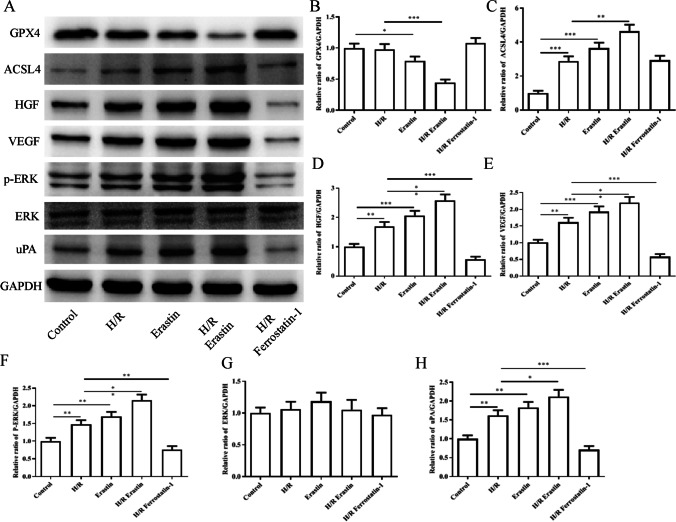
Mechanisms of H/R injury and ferroptosis affecting the function of HCMECs. **A** Western blot was used to detect the protein expression of **B** GPX4, **C** ACSL4, **D** HGF, **E** VEGF, **F** p-ERK, **G** ERK, and **H** uPA. Data are presented as the mean ± SEM (*n* = 3). *, *p* < 0.05; **, *p* < 0.01; ***, *p* < 0.001

To further confirm the findings of the above results, we detected the function-related proteins of ferroptosis, oxidative stress, and mitochondria in HCMECs. As the results show, the erastin administration could activate oxidative stress characterized by decreasing the protein expression of GPX4, whereas ferrostatin-1 treatment could upregulate GPX4 expression (Fig. 3A, B). H/R condition or/and erastin treatment could induce the ferroptosis by promoting ACSL4, but the expression of ACSL4 was reversed in the H/R + ferrostatin-1 group (Fig. 3A, C). As shown in Fig. 3D, E, the levels of HGF and VEGF were increased under H/R condition or/and erastin treatment, which was then decreased in the H/R + ferrostatin-1 group (Fig. 3A, D, E). In addition, the expression of proteins p-ERK and uPA related to cell proliferation and migration was significantly induced under H/R condition or/and erastin treatment, and both were repressed in the H/R + ferrostatin-1 group, while the expression of ERK did not change significantly (Fig. 3A, F, H). These data verified that the effect of H/R injury on cell function is achieved by exacerbating ferroptosis.

### ENPP2 reverses the effect of H/R and ferroptosis on cell migration and angiogenesis in HCMECs

**Fig. 4:**
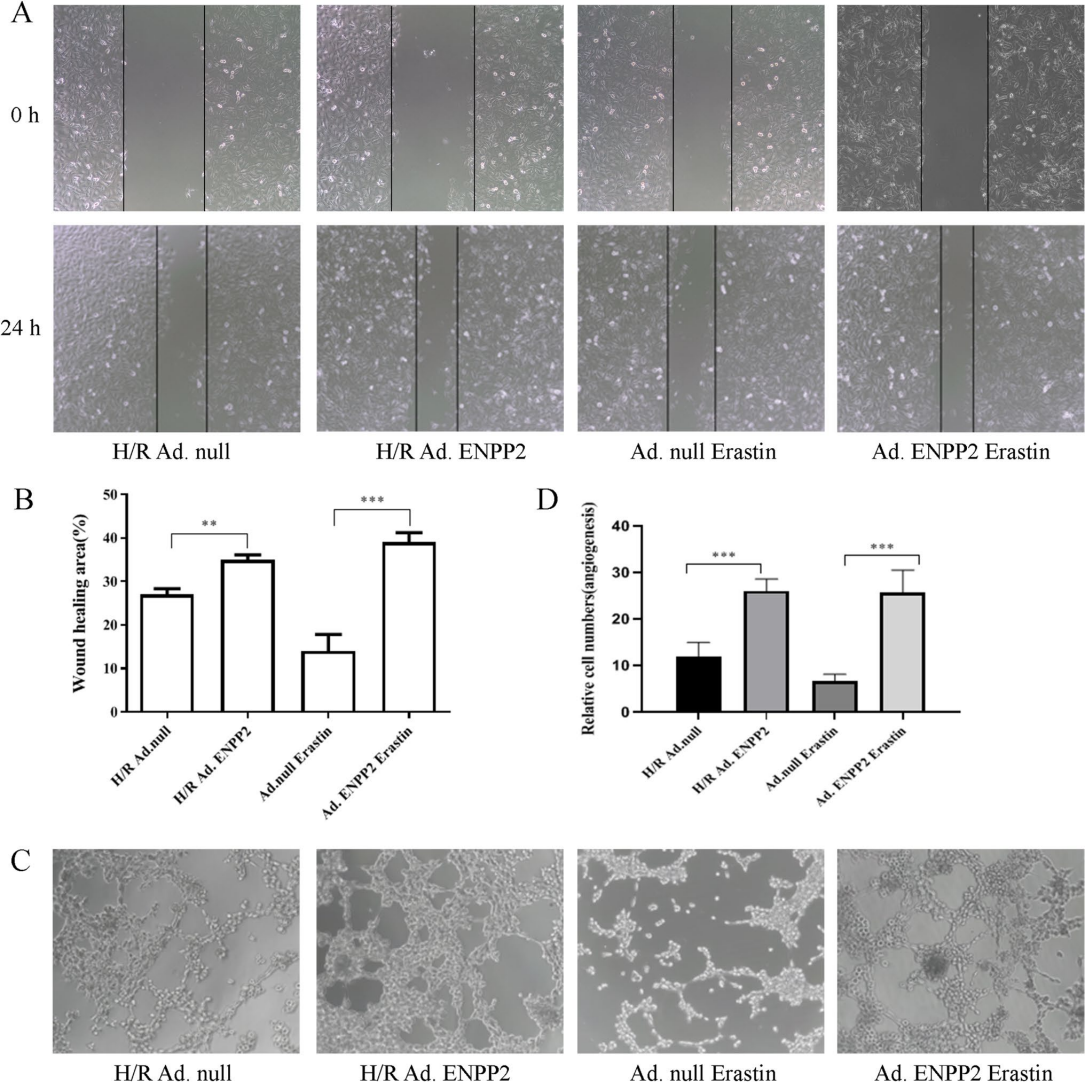
ENPP2 reverses the effect of H/R and ferroptosis on cell migration and angiogenesis in HCMECs. HCMECs with H/R condition or erastin treatment were transfected with Ad. ENPP2 or Ad. null, and the group of which was named as H/R Ad. null, H/R Ad. ENPP2, Ad. null erastin, and Ad. ENPP2 erastin. **A**, **B** The wound healing assay was used to detected the migration ability of HCMECs in each group. **C**, **D** The tube formation assay was used to detect the angiogenesis ability of HCMECs in each group. Data are presented as the mean ± SEM (*n* = 3). *, *p* < 0.05; **, *p* < 0.01; ***, *p* < 0.001

Multiple previous studies have shown that ENPP2 regulates cellular functions, especially cell migration. In order to further explore the molecular mechanism of H/R affecting cell functions by mediating ferroptosis, HCMECs with H/R condition or erastin treatment were transfected with Ad. ENPP2 or Ad. null. The wound-healing test results revealed that compared with the H/R Ad.null or Ad.null erastin group, the cell migration levels in the H/R Ad. ENPP2 and the Ad. ENPP2 erastin groups were significantly increased, indicating that overexpression of ENPP2 can significantly enhance the migration ability of HCMECs (Fig. 4A, B). Similarly, the results of tube formation revealed that in the presence of Ad. ENPP2 transfection group, the tube formation ability of HCMECs was apparently improved compared with the H/R Ad.null or Ad.null erastin group (Fig. 4C, D). The results above suggested that ENPP2 might be involved in the process by which H/R injury inhibits the migration and angiogenesis of HCMECs by regulating ferroptosis.

### ENPP2 alleviates the effects of H/R and ferroptosis on the oxidative stress and mitochondrial function in HCMECs

**Fig. 5:**
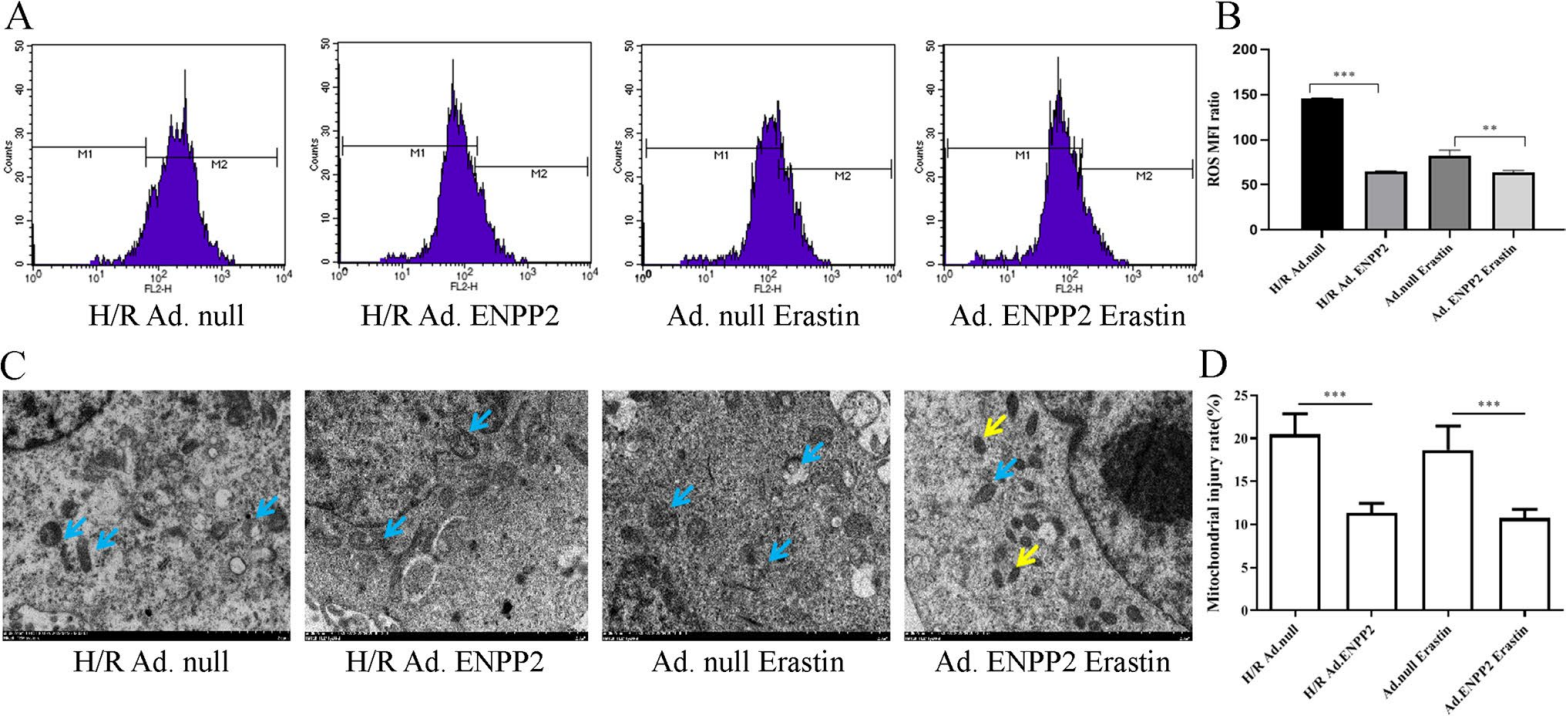
ENPP2 alleviates the effects of H/R and ferroptosis on the oxidative stress and mitochondrial function in HCMECs. **A**, **B** The production of cellular ROS was analyzed by flow cytometry. **C** TEM was used to observe the state of mitochondria in each group of cells; the yellow arrows indicate normal mitochondria and blue arrows indicate swollen cracked mitochondria. **D** Statistical analysis of swollen cracked mitochondria in each group. Data are presented as the mean ± SEM (*n* = 3). *, *p* < 0.05; **, *p* < 0.01; ***, *p* < 0.001

Subsequently, we performed flow cytometry and TEM experiments to detect ROS production and observe mitochondrial morphology in each group of HCMECs. The results of flow cytometry showed that the ROS production was markedly depressed in the H/R Ad. ENPP2 or Ad.ENPP2 erastin group compared with that in the H/R Ad.null or Ad.null erastin group (Fig. 5A, B). Furthermore, compared with the Ad.null transfected group, a marked reduction in mitochondrial swelling and fragmentation was observed in the ENPP2 overexpression group (Fig. 5C, D). These results confirmed that overexpression of ENPP2 could bring down the process by which H/R injury increases ROS production and mitochondrial damage by exacerbating ferroptosis.

### Mechanisms of ENPP2 on hypoxia/reoxygenation (H/R) injury and ferroptosis in HCMECs

**Fig. 6:**
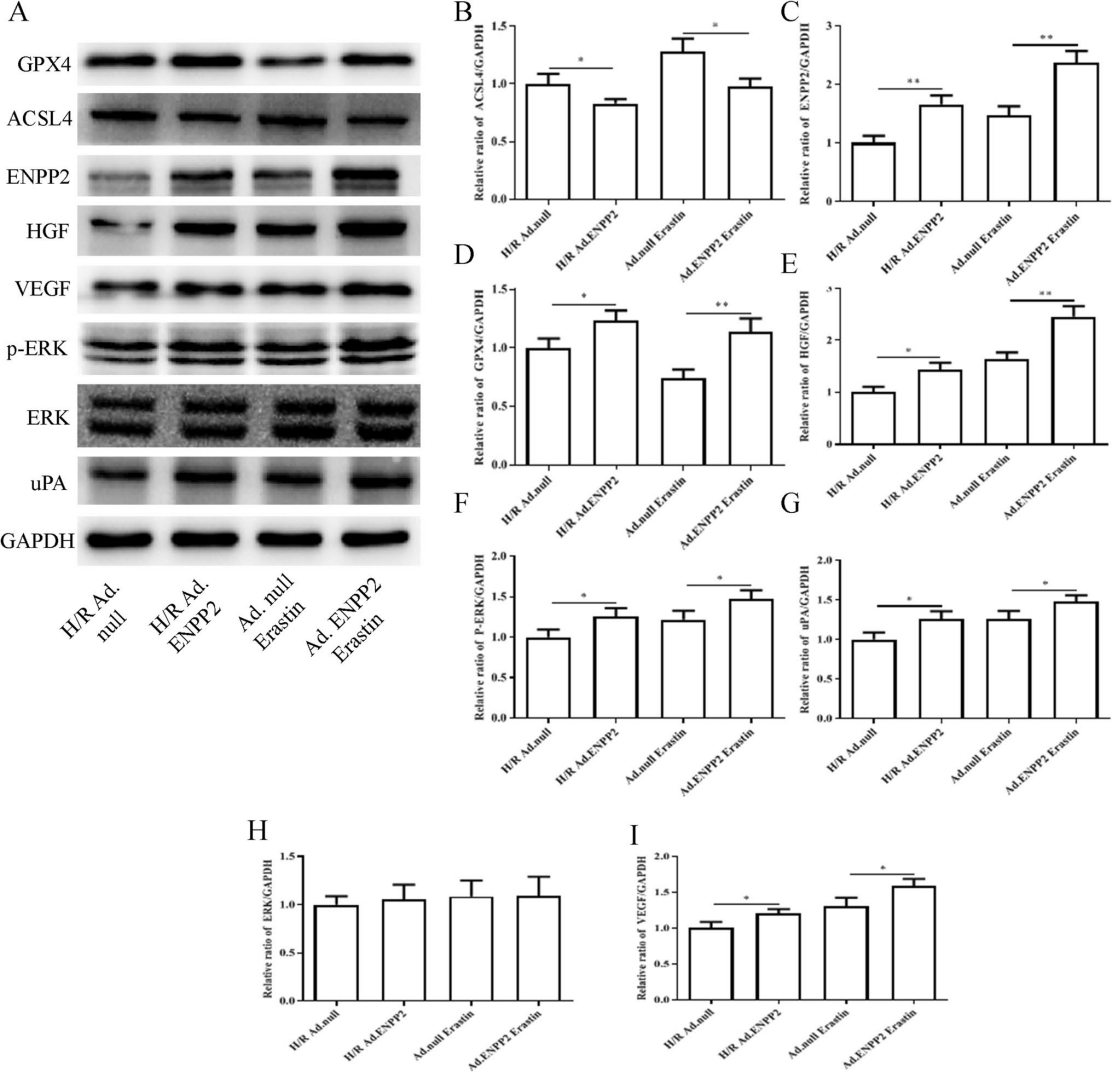
Mechanisms of ENPP2 on hypoxia/reoxygenation (H/R) injury and ferroptosis in HCMECs. **A** Western blot was used to detect the protein expression of **B** GPX4, **C** ENPP2, **D** ACSL4, **E** HGF, **F** VEGF, **G** p-ERK, **H** ERK, and **I** uPA. Data are presented as the mean ± SEM (*n* = 3). *, *p* < 0.05; **, *p* < 0.01; ***, *p* < 0.001

The above results confirmed that overexpression of ENPP2 can alleviate the effects of H/R on cell migration, angiogenesis, oxidative stress and mitochondrial damage by regulating ferroptosis. Next, in order to elucidate the molecular mechanism by which ENPP2 is involved in H/R injury in HCMECs, we examined the expression levels of related proteins in HCMECs. As shown in Fig. 6A, the expression of ENPP2 was up-regulated by Ad. ENPP2 in the H/R Ad. ENPP2 or Ad. ENPP2 erastin group compared with that in the H/R Ad.null or Ad.null erastin group (Fig. 6A, B). Overexpression of ENPP2 could obviously induce the protein expression of GPX4, which is a marker protein of oxidative stress (Fig. 6A, C). In contrast to this, overexpression of ENPP2 could obviously repress the protein expression of ACSL4, indicating that the ferroptosis was alleviated in HCMECs (Fig. 6A, D). The angiogenesis-related proteins HGF and VEGF were significantly elevated in the group with the presence of Ad. ENPP2 (Fig. 6A, E, F). Moreover, after overexpression of ENPP2, the cell proliferation- and migration-related proteins p-ERK and uPA were prominently promoted, but ERK expression did not change significantly (Fig. 6A, G, I). Taken together, these findings suggested that the molecular mechanism by which H/R modulates cell migration, angiogenesis, oxidative stress, and mitochondrial damage in HCMECs by affecting ferroptosis may be achieved by regulating ENPP2.

## Discussion

In recent decades, with the improvement of living standards in China, the prevalence of myocardial ischemia has increased year by year, especially in the middle-aged and elderly population (Zhao et al. 2017). Myocardial I/R injury has many hazards such as increasing endothelial permeability, impairing endothelial barrier function, causing cell swelling, microvascular obstruction, and hindering the blood supply of the heart (Gao et al. 2019; Rios-Navarro et al. 2019). Therefore, effective alleviation of I/R injury is imperative for treating of myocardial ischemia.

Previous studies have revealed that the pathophysiological mechanisms underlying I/R injury might participate in some cellular biochemical processes mainly including oxidative stress, apoptosis, ferroptosis, and energy metabolism (He et al. 2022; Kalogeris, Baines, Krenz, and Korthuis 2012), and the processes above are interrelated and may eventually aggravate cell death directly or indirectly (J. Li et al. 2021). As is known to all, a series of injuries caused by oxidative stress is the main reason for the occurrence and development of I/R injury in different organs such as heart, brain, and liver (Xia, Chen, Fan, and Xue 2014; Xia, Chen, Fan, Xue, and Liu 2015). Oxidative stress is characterized by the excessive production of ROS and the activation of excessive lipid peroxidation, which is precisely the cause of ferroptosis, and leads to the damage of mitochondrial respiratory chain and the imbalance of homeostasis (Park et al. 2021; Wu et al. 2018).

Ferroptosis is an example of an iron-mediated mechanism with pathological effects on the heart. More importantly, emerging studies have reported that ferroptosis induces and aggravates tissue damage following organs ischemia such as cerebral (Wang et al. 2022), renal (Friedmann Angeli et al. 2014), and cardiac (H. Liu et al. 2022), whereas the damage could be rescued by ferrostatin-1, substantiating ferroptosis is a strong contributor to I/R injury (Yu, Guo, Xie, Wang, and Chen 2017). Herein, we investigated the effects of H/R injury on HCMECs by mediating ferroptosis, and H/R model was established with HCMECs followed by the treatment of ferroptosis inducer, erastin, or inhibitor ferrostatin-1. Our results showed that ferrostatin-1 could reverse the effect of H/R injury on HCMECs, including the cell migration, angiogenesis, ROS production, and mitochondrial morphology, indicating that H/R injury was achieved through accelerating the process of ferroptosis.

Subsequently, we further explored the molecular mechanism of H/R injury in HCMECs, and ENPP2 attracted our attention. Enpp2 (exonucleotide pyrophosphatase/phosphodiesterase 2; autophagy), a hemolytic phospholipase D from the arterial wall, encourages atherogenic monocyte adhesion by inducing lysophosphatidic acid (LPAS). ENPP2 regulates a variety of biological functions through the homologous G protein-coupled receptor LPAR1-6. Enpp2 promotes tumor cell dispersal, migration, and metastasis through LPAR1 (Auciello et al. 2019; Lin et al. 2019) and T cell motility through LPAR2 (Knowlden et al. 2014; Takeda et al. 2016). Most notably, ENPP2 protects cardiomyocytes from erastin-induced ferroptosis, and ENPP2 overexpression notably promotes migration and proliferation and suppresses erastin-induced ferroptosis of H9c2 cells (Bai et al. 2018). So far, in our study findings, we demonstrated that ENPP2 overexpression had an ability to prevent the development of H/R injury through blocking ferroptosis.

To sum up, our study identified that the injury of myocardial I/R to HCMECs might concern cell migration ability, cellular oxidative stress, ferroptosis level, and mitochondrial function. Moreover, we also emphasize that ENPP2 has been commonly used in the study on tumor progression previously, but our data show that it can also participate in myocardial I/R injury by regulating the level of oxidative stress and ferroptosis, but the specific downstream targets and related signaling pathways need to be further studied. As a supplement to previous studies, our findings establish a theoretical foundation for clinical treatment of myocardial I/R.
